# Relationship of porcine *IGF2 *imprinting status to DNA methylation at the *H19 *DMD and the *IGF2 *DMRs 1 and 2

**DOI:** 10.1186/1471-2156-12-47

**Published:** 2011-05-17

**Authors:** Martin H Braunschweig, Marta Owczarek-Lipska, Nasikhat Stahlberger-Saitbekova

**Affiliations:** 1 Institute of Genetics, Vetsuisse Faculty, University of Bern, 3001 Berne, Switzerland

## Abstract

**Background:**

Porcine *IGF2 *and the *H19 *genes are imprinted. The *IGF2 *is paternally expressed, while the *H19 *gene is maternally expressed. Extensive studies in mice established a boundary model indicating that the H19 differentially methylated domain (DMD) controls, upon binding with the CTCF protein, reciprocal imprinting of the *IGF2 *and the *H19 *genes. *IGF2 *transcription is tissue and development specific involving the use of 4 promoters. In the liver of adult Large White boars *IGF2 *is expressed from both parental alleles, whereas in skeletal muscle and kidney tissues we observed variable relaxation of *IGF2 *imprinting. We hypothesized that *IGF2 *expression from both paternal alleles and relaxation of *IGF2 *imprinting is reflected in differences in DNA methylation patterns at the *H19 *DMD and *IGF2 *differentially methylated regions 1 and 2 (DMR1 and DMR2).

**Results:**

Bisulfite sequencing analysis did not show any differences in DNA methylation at the three porcine CTCF binding sites in the *H19 *DMD between liver, muscle and kidney tissues of adult pigs. A DNA methylation analysis using methyl-sensitive restriction endonuclease *Sac*II and 'hot-stop' PCR gave consistent results with those from the bisulfite sequencing analysis. We found that porcine *H19 *DMD is distinctly differentially methylated, at least for the region formally confirmed by two SNPs, in liver, skeletal muscle and kidney of foetal, newborn and adult pigs, independent of the combined imprinting status of all *IGF2 *expressed transcripts. DNA methylation at CpG sites in DMR1 of foetal liver was significantly lower than in the adult liver due to the presence of hypomethylated molecules. An allele specific analysis was performed for *IGF2 *DMR2 using a SNP in the *IGF2 *3'-UTR. The maternal *IGF2 *DMR2 of foetal and newborn liver revealed a higher DNA methylation content compared to the respective paternal allele.

**Conclusions:**

Our results indicate that the *IGF2 *imprinting status is transcript-specific. Biallelic *IGF2 *expression in adult porcine liver and relaxation of *IGF2 *imprinting in porcine muscle were a common feature. These results were consistent with the *IGF2 *promoter P1 usage in adult liver and *IGF2 *promoter P2, P3 and P4 usages in muscle. The results showed further that bialellic *IGF2 *expression in liver and relaxation of imprinting in muscle and kidney were not associated with DNA methylation variation at and around at least one CTCF binding site in *H19 *DMD. The imprinting status in adult liver, muscle and kidney tissues were also not reflected in the methylation patterns of *IGF2 *DMRs 1 and 2.

## Background

Porcine insulin-like growth factor 2 (*IGF2*) and *H19 *genes are reciprocally imprinted in most tissues. In mice, these two genes share common endodermal and mesodermal enhancers and the mouse *Igf2 *gene is also paternally expressed in most tissues whereas the *H19 *gene is maternally transcribed [1-3]. Mice lacking the *Igf2 *gene weighed about 40% less than their litter mates [4]. The *H19 *gene expresses a non-protein-coding RNA and is located 88.1 kb downstream of *IGF2 *[5,6]. Recently, it was found that *H19 *transcripts can function as microRNA precursors [7].

The pig *INS-IGF2-H19 *imprinting cluster is highly homologous to the corresponding human gene cluster and is thus a good model to study epigenetic mechanisms [5]. Recently, a quantitative trait nucleotide (QTN) at position *IGF2*-intron3-3072 was identified and various antisense transcripts originate from the paternal allele demonstrating the complex transcription from this gene [8,9].

An extensive number of studies have been conducted to elucidate the epigenetic mechanisms of *IGF2 *and *H19 *which are thought to be co-ordinately regulated, both in terms of their expression patterns and their reciprocal imprinting (for review see [3]). It was shown by deletions in mice that a region of paternal-specific DNA methylation (differentially methylated domain, DMD) upstream of *H19 *is an epigenetic mark required for imprinting of *IGF2 *and *H19 *[10,11]. Bell and Felsenfeld [12] reported that activity of *H19 *DMD depends upon the vertebrate eleven-zinc finger protein CTCF that binds to this DMD and mediates the function of the boundary/insulator element. They also found that methylated CpG sites at the CTCF binding site abolished binding *in vitro*. Based on this finding Bell and Felsenfeld [12] developed a model explaining the reciprocal imprinting. On the maternal allele the enhancer downstream of *H19 *has no access to the *IGF2 *promoters due to the boundary function of CTCF proteins bound to the unmethylated DMD whereas the *H19 *gene can still be transcribed. On the paternal allele DNA methylation at the *H19 *DMD eradicates the boundary function which leads to *IGF2 *gene transcription and silencing of the *H19 *gene. These findings were made simultaneously using transgenic mice and cell culture and contributed to establish the boundary model [13]. More recently it was demonstrated that differentially methylated regions in the mouse *Igf2 *and *H19 *genes interact in an epigenetically regulated manner that partition maternal and paternal alleles into distinct loops. The maternal allele *H19 *DMD interacts with *Igf2 *DMR1 allowing maternal *H19 *to be expressed while the paternal *H19 *DMD interacts with *Igf2 *DMR2, allowing *Igf2 *to be expressed and leaves the *H19 *gene silent. This model was named the chromatin loop model [14].

**Figure 1:**
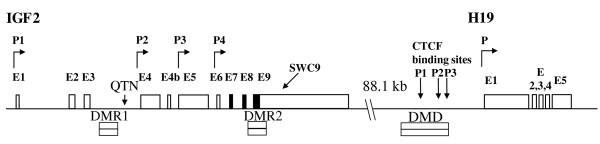
**Schematic drawing of the *IGF2 *and *H19 *gene loci**. *IGF2 *exons (E), promoters (P1 to P4 and P), differentially methylated regions (DMR1 and 2), the *H19 *differentially methylated domain (DMD), the quantitative trait nucleotide (QTN) and the microsatellite SWC9 are indicated. CTCF binding sites in P1, P2 and P3 repeats are indicated in the DMD.

A DMD between -1.1 and -2.6 kb upstream of the *H19 *transcription start site has also been found to regulate porcine reciprocal transcription of *H19 *and *IGF2 *via a CTCF protein that binds to the unmethylated maternal allele (Figure 1). In the pig *H19 *DMD, three CTCF-binding motifs in P1, P2 and P3 repeats were identified by sequence homology to the human *H19 *DMD [5]. Relaxation of *IGF2 *imprinting was observed in skeletal muscle of 4 month old pigs compared to nearly complete monoallelic expression in prenatal skeletal muscle [8]. Wrzeska et al. [15] reported biallelic *IGF2 *expression in adult porcine liver and brain and monoallelic *IGF2 *expression in muscle and kidney tissues of adult pigs. Recently, it was reported that *IGF2 *transcripts from promoter P1 are also biallelically expressed in tissues of week-old pigs whereas in most other tissues, including skeletal muscle and kidney, *IGF2 *was monoallelically expressed [16]. Biallelic expression of *Igf2*/*IGF2 *has been reported for choroids plexus and leptomeninges of the mouse as well as in postnatal and normal adult human liver [1,17,18]. A recent report by Wu et al. [19] confirmed biallelic *IGF2 *expression in normal human liver and showed a medium-methylated *H19 *DMD profile with a hypermethylated paternal and a hypomethylated maternal allele.

In an ongoing study we investigated the combined imprinting status of all *IGF2 *expressed transcripts in liver, skeletal muscle and kidney tissues of adult boars. We hypothesized that an alteration in *IGF2 *imprinting status might be reflected in DNA methylation variations at differentially methylated regions as suggested by the boundary and chromatin loop models. To test this hypothesis we studied the association between the *IGF2 *imprinting status in three different tissue samples of six boars and their DNA methylation of *H19 *DMD, *IGF2 *DMR1 and DMR2 (Figure 1). Furthermore, we included samples from two foetus and two newborn pigs in order to examine *IGF2 *imprinting and DNA methylation at these differentially methylated regions during development. We were curious to see whether the imprinting status of *IGF2 *in liver, skeletal muscle and kidney at different developmental stages is also reflected in the DNA methylation patterns of *H19 *DMD and in particular at CTCF binding sites as well as in *IGF2 *DMR1 and *IGF2 *DMR2.

## Results

### Imprinting status of *IGF2*

**Figure 2:**
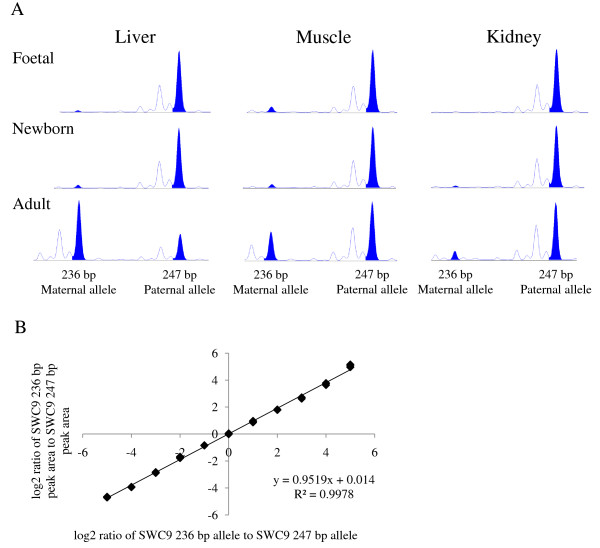
***SWC9 *microsatellite marker capillary profiles of cDNA from pig liver, skeletal muscle and kidney tissues and a standard curve of a dilution series of the alternative alleles**. **A **The 247 bp allele is paternally inherited and the size of the peak area for the maternal 236 bp allele is indicative for the level of *IGF2 *imprinting relaxation. The cDNA profiles from adult liver tissues show a similarity to biallelic *IGF2 *expression while those of cDNA from adult skeletal muscle and kidney indicate relaxation of imprinting. **B **Standard curve of mixed DNA from respective homozygote individuals with 11 different ratios (see also in the section methods).

We used the *SWC9 *microsatellite marker with alleles 236 bp and 247 bp to investigate the combined imprinting status of *IGF2 *gene expression in liver, skeletal muscle and kidney (Figure 1). The *SWC9 *microsatellite is located in the 3' UTR of the *IGF2 *gene and its sequence is common to all *IGF2 *transcripts originating from different promoter usages in a specific tissue and developmental stages. We investigated two pig foetuses, two newborn piglets and six boars that were heterozygous for the *SWC9 *marker. All but one boar received the *SWC9 *allele 247 bp from their fathers and the *SWC9 *236 bp allele from their mothers. We established a standard curve with a dilution series of DNA from alternative homozygous individuals for the *SWC9 *marker [20]. As a measure for the imprinting status we calculated the log2 ratio of the *SWC9 *236 bp peak area to the *SWC9 *247 bp peak area and calculated the corresponding ratio of *SWC9 *236 bp allele to *SWC9 *247 bp based on the standard curve (Figure 2B). In Table 1 the ratios of allelic *IGF2 *expression for the different tissues and development stages are shown. This experiment showed that the imprinting status of *IGF2 *changes from paternal expression in the foetal liver to *IGF2 *expression from both parental alleles in the adult liver. Paternal *IGF2 *gene expression is mostly maintained in kidney tissue during development. In muscle tissue a trend of relaxation of imprinting during development is indicated. The considerable variation of these ratios is mostly explained by the small sample size and a few samples that substantially deviate from the mean but were nonetheless kept in the analysis. *IGF2 *imprinting in liver, muscle and kidney of foetal, newborn and adult pigs is visualized by the *SWC9 *microsatellite profiles in Figure 2A.

**Table 1:** Imprinting status in different pig tissues and for three development stages expressed as ratio of paternal to maternal *IGF2 *gene expression.

Tissue	Ratio paternal to maternal gene expression (± SD)
Adult liver (N = 6)	4.2 (± 6.2)
Newborn liver (N = 2)	21.3 (± 0.01)
Foetal liver (N = 2)	Paternal expression
Adult muscle (N = 6)	9.2 (± 6.2)
Newborn muscle (N = 2)	19.9 (± 6.0)
Foetal muscle (N = 2)	Paternal expression (N = 1) and 14 (N = 1))
Adult kidney (N = 6)	22.9 (± 10.2)
Newborn kidney (N = 1)	34.0
Foetal kidney (N = 2)	Paternal expression

The observed biallelic *IGF2 *expression in these adult boars' liver is in agreement with previous reports showing biallelic expression of *IGF2 *in the livers of humans and pigs [15-19]. RT-PCR analysis of adult liver cDNA indicated that all 4 *IGF2 *promoters were used whereas cDNA from muscle and kidney revealed very low level of products after 40 PCR cycles for transcripts from promoter P1 and high level of products for transcripts from promoter P2, P3, and P4 (data not shown). This result is in agreement with previous findings from Amarger et al. and Li et al. [5,16], however, from previous Northern blot analysis it is known that promoter P1 is predominantly used in adult pig liver and transcripts from promoter P2, P3 and P4 were not detected. The RT-PCR analysis of microsatellite *SWC9 *is a semi-quantitative approach to investigate *IGF2 *imprinting status and the results conclusively indicate that *IGF2 *imprinting is reversed similarly to biallelic expression in adult liver and it is relaxed to different degrees in muscle and kidney tissues during development.

### Bisulfite sequencing analysis of *H19 *DMD

**Figure 3:**
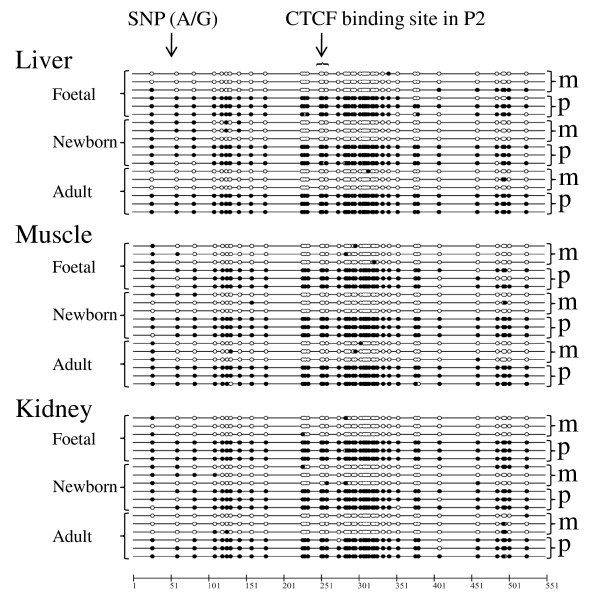
**Bisulfite sequencing analysis of the CTCF consensus binding site in the P2 repeat of *H19 *DMD**. Filled circles are methylated and open circles are unmethylated cytosines at CpG sites. Maternal and paternal origins are indicated by m and p, respectively. The position of the CTCF binding site in the P2 repeat and of the SNP (AY044827.1:g.32619G>A) are given.

Bisulfite sequencing of porcine *H19 *DMD indicated that the region was indeed differentially methylated from about -1 kb to -3 kb from the start site of *H19 *exon 1 (AY044827.1). We sequenced bisulfite treated DNA from four overlapping PCR fragments derived from adult liver, skeletal muscle and kidney DNA and found distinct hyper- and hypomethylated molecules in each of the four fragments. This strongly suggests that *H19 *DMD extended at least over this region (PCR primers *H19*_DMD_1 to *H19*_DMD_4, Additional file 1 Table S1). In the two PCR fragments spanned by PCR primer pairs (*H19*_DMD_2 and *H19*_DMD_3) two SNPs (AY044827.1:g.32530C>T and AY044827.1:g.32619G>A) were identified and thus the parental alleles deduced which confirmed the DMD. This result is in perfect agreement with the boundary model which explains the mechanism by which CTCF's binding at the *H19 *DMD mediates chromatin insulator function and thus maintains the reciprocal imprinting of the *IGF2 *and *H19 *genes. We have analysed DNA methylation and in particular CpG sites at the CTCF binding site in the P2 repeat in liver, muscle and kidney tissues of foetal, newborn and adult pigs [5]. In Figure 3 DNA methylation patterns of the bisulfite sequencing analysis are shown as a 'lollipop' graphic. There is no apparent difference in DNA methylation at these three CpG sites within the CTCF binding site P2 between liver, skeletal muscle and kidney tissues in all development stages. Furthermore, a bisulfite sequencing analysis from 7 to 13 clones from each of the three tissues liver, kidney and muscle of three adult boars indicated hyper- and hypomethylatd CTCF binding sites in P1 and P3 repeats. This result is consistent with a medium-methylated *H19 *DMD profile, although we did not find a DNA polymorphism to determine the parental origin of these clones (data not shown). Sanger sequencing of bisulfite treated DNA molecules is laborious and only a relatively small number of molecules can be sequenced. There may also be heteroduplex artifacts present during PCR amplification of bisulfite treated DNA molecules because the bacterial host's mismatch repair system can convert paternal and maternal alleles into single hybrid sequences during cloning. To account for these drawbacks we performed a DNA methylation analysis using *Sac*II and nonradioactive 'hot-stop' PCR [21]. This assay discriminates between DNA methylation at maternal and paternal derived alleles at the CTCF binding site in the P2 repeat.

### DNA methylation analysis using methyl-sensitive restriction endonuclease

**Figure 4:**
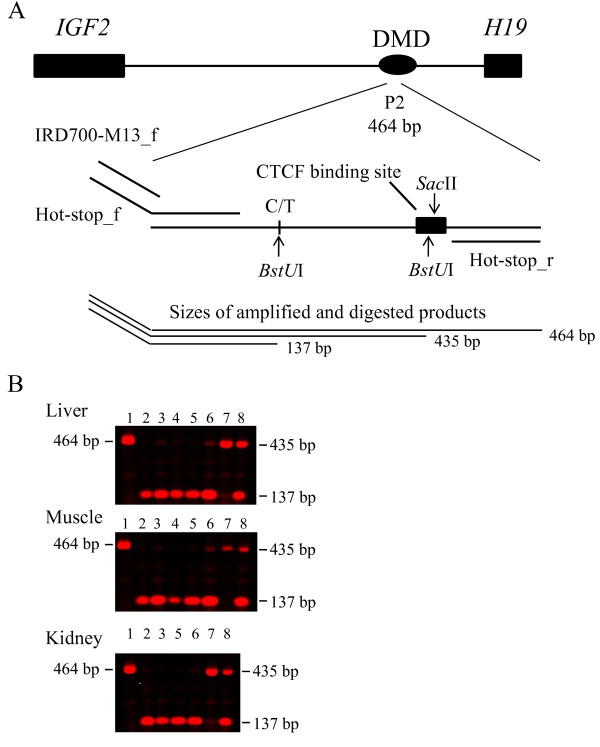
**Methylation-sensitive restriction endonuclease *Sac*II digest of genomic DNA, 'hot-stop' PCR and subsequent *BstU*I digest to discriminate between the maternal and paternal alleles**. **A **Schematic representation of the DNA methylation analysis using methyl-sensitive restriction endonuclease *Sac*II and 'hot-stop' PCR in the *H19 *DMD containing the P2 repeat. The recognition site of *Sac*II, 'hot-stop' PCR primers and the *BstU*I recognition sites are indicated. **B **Scanned agarose gel by infrared fluorescent detection. Lane 1 is an undigested 464 bp product that is 29 bp longer than the digested longer allele since there is a second *BstU*I restriction site. Lanes 2 and 3 show 137 bp fragments from foetal DNA indicating paternal hypermethylation and maternal hypomethylation at the CTCF P2 binding site. Lanes 4 and 5 are the 137 bp DNA fragments from newborn tissues and lanes 6 and 7 (137 bp and 435 bp fragments, the later having the alternative paternal allele methylated) are amplified DNA fragments from adult tissues. Also in lanes 6 and 7 paternal hypermethylation and maternal hypomethylation of the CTCF P2 binding sites are demonstrated. In lane 8 a control result of a 1:1 mix of DNA from respective homozygous CC and TT individuals for the AY044827.1:g.32530.C>T polymorphism is shown.

In Figure 4 the assay of the DNA methylation analysis using a methyl-sensitive restriction endonuclease is shown. *Sac*II recognizes the CTCF binding site in the P2 repeat and does not cleave if one or both of the two CpG sites are methylated, i.e. the amplified products represent at least one methylated CpG at the binding site. Paternal and maternal alleles are discriminated by a restriction site for *BstU*I. This assay detects the ratio between paternal and maternal CpG methylation at the P2 repeat in *H19 *DMD. The proportions of unmethylated CpG sites in the CTCF binding site in the P2 repeat are not accounted for by this method. In lane 1 an undigested 464 bp PCR product is shown that is 29 bp longer than the digested longer allele since there is a second *BstU*I restriction site located in the CTCF binding site. Lanes 2 and 3 show the 137 bp fragment from foetal tissues indicating paternal hypermethylation and maternal hypomethylation at the CTCF P2 binding site. Also in newborn pig tissues (lanes 4 and 5, fragments of 137 bp) and in the tissues of adult pigs (lanes 6 and 7, 137 bp and 435 bp, the later having the alternative paternal allele methylated) paternal hypermethylation and maternal hypomethylation at this CTCF P2 binding sites is suggested. In lane 8 a control result of a 1:1 mix of DNA from respective homozygous CC and TT boars for the AY044827.1:g.32530.C>T polymorphism is shown. The results of this assay consistently indicate paternal hypermethylation and maternal hypomethylation at this CTCF binding site in the *H19 *DMD in the three tissues and for the three development stages.

### Bisulfite sequencing analysis of *IGF2 *DMR1

DNA polymorphisms that allowed the deduction of the parental origin of alleles in the *IGF2 *DMR1 were not found. Nevertheless we used bisulfite sequencing to analyse a considerable number of clones to search for specific DNA methylation patterns both between the three tissue samples and during development, which are supposed to be associated with the *IGF2 *imprinting status. We compared DNA from between 7 and 37 single clones per tissue and developmental stage and could not find any significant difference between DMR1 methylation in muscle and kidney tissues within foetal, newborn and adult individuals (Wilcoxon two-sample test, two-sided).

**Figure 5:**
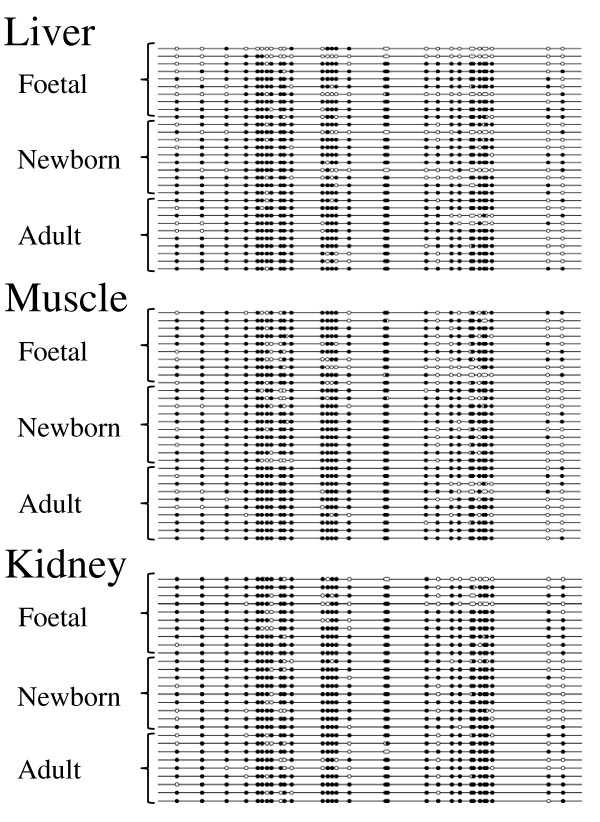
**Bisulfite sequencing analysis of the *IGF2 *DMR1 in porcine liver, muscle and kidney tissues**. Filled circles are methylated and open circles are unmethylated cytosins at CpG sites. The clones are a random selection from all individuals and different tissues. DMR1 is hypermethylated but hypomethylated clones in foetal liver were also detected. The parental origin of the clones is unknown due to the lack of an informative SNP.

There was a significant difference in DNA methylation at DMR1 CpG sites between 22 clones from the foetal liver compared to that of 33 clones from the adult pig liver (*P *< 0.01, Wilcoxon two-sample test, two-sided). A suggestive difference in DNA methylation at DMR1 was found between 22 clones from foetal liver tissues and 13 clones from livers of two newborn piglets (*P *= 0.06). A closer inspection of the data showed a small fraction of hypomethylated DMR1 clones in the foetal liver. This result must be interpreted cautiously due to the small sample number. DMR1 was hypermethylated with over 70% DNA methylation in all tissue samples except for foetal liver and the foetal muscle, which had 66% (22 clones) and 68% (22 clones) DNA methylation, respectively. Representative lollipop diagrams are given in Figure 5 for all tissues and the three developmental stages. In summary, the DMR1 at the *IGF2 *locus was largely hypermethylated with some indication of hypomethylated DNA molecules in foetal liver.

### Bisulfite sequencing analysis of *IGF2 *DMR2

**Figure 6:**
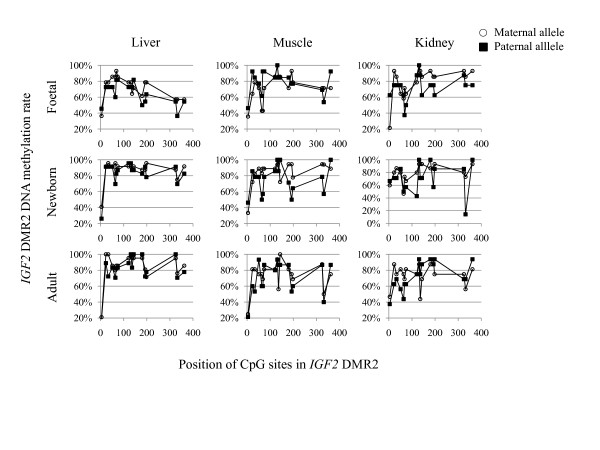
**The mean percentage of DNA methylation at each CpG site of maternal and paternal *IGF2 *DMR2 clones are shown for liver, muscle and kidney and three development stages foetal, newborn and adult**. Profiles were similar for both parental alleles and the three different tissue samples. For most of the CpG sites a moderate hypermethylation was found. A minor higher DNA methylation content was observed on the maternal allele in the foetal and newborn liver as compared to the paternal allele. The *IGF2*-exon9-612A>T SNP is located downstream of the 17^th ^CpG site and not indicated.

We used the *IGF2*-exon9-612A>T SNP to determine the parental origin of DMR2 molecules from the bisulfite sequence analysis. In Figure 6 maternal and paternal profiles of the DNA methylation at each of the 17 CpG sites are shown for three tissues and three developmental stages. Overall results showed hypermethylation of both parental DMR2 alleles. We focused on differences in DNA methylation of the two parental alleles in three tissues and during development. We found significantly higher DNA methylation along the 17 CpG sites on the maternal allele in foetal (14 maternal clones versus 11 paternal clones) and newborn liver (24 maternal clones versus 23 paternal clones) compared to the paternal allele (*P *< 0.05, Wilcoxon two-sample test, two-sided). However, it should be emphasised that both alleles were hypermethylated with 71% and 64% in foetal and 87% and 82% in the newborn liver for the respective maternal and the paternal alleles. There was a tendency that the maternal allele was also more methylated in foetal and newborn muscle and kidney tissues compared to the paternal allele. No significant differences in DNA methylation were observed between the parental alleles in the adult tissues.

## Discussion

Van Laere et al. [8] and Wrzeska et al. [15] showed relaxation of imprinting in skeletal muscle tissues of 4 month old pigs and exclusive paternal *IGF2 *expression in the tissues of adult pigs' skeletal muscle, respectively. It is important to point out that insufficient PCR cycles, or template, may lead to the product arising from the maternal allele to go undetected, especially in the muscle and kidney, where relaxation of imprinting seems to increase with aging (Figure 2). This might be a reason for the conflicting data found by Van Laere et al. [8] and Wrzeska et al. [15]. In a comprehensive study the imprinting status of *IGF2 *and *H19 *were determined in 13 tissue samples of week-old piglets [16]. Li et al. (2008) [16] found biallelic *IGF2 *expression from promoter P1 in heart, liver, brain, lung, kidney, stomach, pancreas, thymus, tongue, muscle, bladder, spleen, and placenta tissues of week-old pigs. Their RT-PCR analysis of microsatellite *SWC9 *in these 13 different tissues revealed, however, exclusive or nearly exclusive paternal *IGF2 *expression. Furthermore, their real-time PCR analysis of *IGF2 *exon 2 originating from promoter P1 and of *IGF2 *exon 9, that is common for all *IGF2 *transcripts, resulted in roughly 33% and 10% promoter P1 *IGF2 *transcription relative to total *IGF2 *transcription in brain and placenta, respectively. These results are in agreement with the data presented for *IGF2 *imprinting in liver and suggest that transcription from the *IGF2 *promoter P1 may be regulated by other mechanisms than that from promoters P2, P3 and P4. Together these data emphasize the promoter-specific *IGF2 *imprinting status in different tissues and during development [16]. Biallelic expression was also observed in the liver and brain of 6-month-old lambs but not in their kidneys [22].

*H19 *DMD was paternally hypermethylated and maternally hypomethylated in liver, muscle and kidney of all three developmental stages independent of the combined imprinting status of all *IGF2 *expressed transcripts. This finding challenges the boundary model [12,13] postulating that the vertebrate eleven-zinc finger protein CTCF binds the maternal unmethylated *H19 *DMD insulating the upstream *IGF2 *promoters from enhancers downstream of *H19*. On the paternal allele the methylated DMD abolishes CTCF binding and enhancers 3' of *H19 *have access to *IGF2 *promoters. Our results demonstrate that *IGF2 *is expressed from both alleles, mainly in adult liver and, to a much lesser extent in skeletal muscle and kidney, although *H19 *DMD is indeed differentially methylated. Histone modifications might still cause these effects but if so, they would be independent of DNA methylation. Investigations of mouse *Igf2 *DMR 1 and 2 led to a model of parent-specific chromatin loops that regulate *Igf2 *imprinting [14]. DNA methylation at DMR 1 and 2 in our samples does not support a parent-specific chromatin loop model regulating *IGF2 *imprinting in pig. It remains inconclusive if subtle differences in DNA methylation in DMR1 between foetal and adult liver and that between the parental alleles in DMR2 of foetal and newborn liver are involved in the control of the *IGF2 *imprinting status.

## Conclusions

From our imprinting and DNA methylation analyses we conclude, firstly, that *IGF2 *expression from both parental alleles in adult porcine liver and relaxation of *IGF2 *imprinting in adult porcine muscle are not associated with DNA methylation variation at and around at least one CTCF binding site in *H19 *DMD. Secondly, the lower DNA methylation content in DMR1 in foetal liver, as compared to adult liver, should be evaluated on molecules from which the parental origin could be established. Thirdly, similar to DMR1 porcine DMR2 is hypermethylated on both parental alleles rather than differentially methylated, as observed for *H19 *DMD. Furthermore, the maternal DMR2 allele was more methylated in foetal and newborn animals when compared to the respective paternal allele. Finally, the transition of *IGF2 *imprinting in foetal liver to *IGF2 *expression from both alleles in adult liver may inherent new mechanisms involved in *IGF2 *imprinting regulation and provides a promising subject for further study.

## Methods

### Animals

From a collection of Swiss Large White pigs 2 male foetus, 1 female and 1 male newborn and 6 adult boars were selected based on their heterozygosity for the microsatellite marker *SWC9 *located in the 3'-UTR of the *IGF2 *gene.

### DNA and RNA isolation and first strand cDNA synthesis

DNA was isolated from liver, skeletal muscle and kidney tissues using the DNeasy Blood & Tissue Kit from Qiagen (Hombrechtikon, Switzerland). RNA extraction was performed with Trizol^® ^Reagent according to the manufacturer's protocol (Invitrogen, Lucerne, Switzerland). Total RNA was digested with RNase-Free DNAse I according to the supplier's instructions (Ambion, Rotkreuz, Switzerland). DNA free RNA was reverse transcribed using a First-Strand cDNA Synthesis Kit (GE Healthcare, Glattbrug, Zurich) and products were subsequently purified with QIAquick columns (Qiagen).

### Imprinting status

Imprinting status was investigated by means of the *SWC9 *microsatellite marker located in the 3'-UTR of the *IGF2 *gene (Figure 1). The *SWC9 *microsatellite marker was amplified using Qiagen's Multiplex PCR Master Mix including a FAM labeled forward primer. PCR was performed with an initial step at 94°C for 15 minutes and 34 cycles of a denaturation step at 94°C for 30 seconds, an annealing step at 60°C for 1 and a half minutes and an elongation step at 72°C for 1 minute, and a final elongation for 10 minutes. All samples including the standard samples were analysed in triplicates and subjected to the same PCR run in a 96 well plate. Sequences of the PCR primers are shown in Supplementary Table 1. Following PCR 1 μl of each reaction was combined with 10 μl of genotyping mix (980 μl of HiDi formamide and 20 μl of GeneScanTM-500 LIZTM Size Standard (Applied Biosystems, Rotkreuz, Switzerland). The mixture was denatured for 2 min., chilled on ice and loaded on an ABI 3730 capillary sequencer (Applied Biosystems). Data was analyzed using GeneMapper software version 4.0 (Applied Biosystems). A standard dilution series was established based on the peak areas of mixed DNA samples from two homozygous individuals for the respective SWC9 236 and SWC9 247 alleles. We used ratios of 32:1, 16:1, 8:1, 4:1, 2:1, 1:1, 1:2, 1:4, 1:8, 1:16, and 1:32 of the respective SWC9 236 and SWC9 247 homozygous DNA samples. The ratio of the peak areas was used to calculate the ratio between the SWC9 alleles 236 and 247 according to the formula y = 0.9519x + 0.014 Figure 2 and[20].

### Bisulfite sequencing

DNA was converted with the EpiTect Bisulfite kit according to the supplier's manual (Qiagen). Bisulfite-conversion-based methylation PCR primers were designed with the program Methprimer http://www.urogene.org/methprimer/index.html and in the case of *IGF2*_DMR2 with Methyl Primer Express (ABI). Primer sequences and product sizes of the 4 fragments covering the *H19 *DMD (*H19*_DMD_1, *H19*_DMD_2, *H19*_DMD_3, *H19*_DMD_4), the *IGF2 *DMR1 (*IGF2*_DMR1) and the *IGF2 *DMR2 (*IGF2*_DMR2) is shown in Additional file 1 Table S1. PCR was performed with the Multiplex PCR Master Mix and products from liver, skeletal muscle and kidney were cloned (TOPO TA Cloning Kit (Invitrogen). White colonies were picked diluted in 50 μl water and amplified with the illustra™ TempliPhi amplification kit (GE Healthcare) and sequenced on an ABI 3730 capillary sequencer (Applied Biosystems). Bisulfite sequencing analysis was performed with the programs BiQ Analyzer http://biq-analyzer.bioinf.mpi-sb.mpg.de/ and MethTools http://genome.fli-leibniz.de/methtools/.

### DNA methylation analysis using methyl-sensitive restriction endonuclease *Sac*II and 'hot-stop' PCR

About 100 ng genomic DNA was digested over night with the methyl-sensitive *Sac*II endonuclease (New England BioLabs, Allschwil, Switzerland). CpG methylation of the recognition site CCGCGG inhibits digestion. A *Sac*II recognition site is present in a CTCF binding site 5' upstream of *H19 *and is referred to pig repeat P2 [5]. We previously re-sequenced the pig imprinting control region (ICR) containing the three pig CTCF binding sites upstream of *H19*. By this means we identified two SNPs, one at position AY044827.1:g.32530C>T and the other at position AY044827.1:g.32619G>A. PCR primers ('hot-stop') were designed which encompass the CTCF binding site in P2 and the two SNPs (Supplemented Table 1). The forward primer is tailed with a M13 forward sequence. An aliquot of the *Sac*II digested DNA was PCR amplified for each sample using these primers by a 'hot-stop' PCR procedure for linear quantification of allele ratios [23]. The 'hot-stop' PCR was performed with Multiplex PCR Master Mix (Qiagen) for 35 cycles with an initial denaturation step at 94°C for 15 min followed by denaturation at 94°C for 30 sec, an annealing temperature of 55°C for 30 sec, an annealing temperature of 68°C for 30 sec and an elongation step at 72°C for 30 sec. The PCR was then paused after these 34 cycles at 72°C and 0.2 μM IRDye™700 labeled M13 primers was added to the reaction. The PCR was then resumed for an additional cycle and a final elongation step at 72°C for 10 min.

The PCR product contains a common 5'...CGCG...3' recognition site for *BstU*I (New England BioLabs) and a second that includes the AY044827.1:g.32530C>T SNP which was used to discriminate the parental origin of the alleles in this fragment of *H19 *DMD. An aliquot of the 'hot-stop' PCR was subsequently digested over night with *BstU*I according to the supplier's recommendation. The digested products were separated on a 1.5% agarose gel and scanned on an Odyssey Infrared Imaging System according to LI-COR's instruction (LI-COR Biosciences, Bad Homburg, Germany). Bands were visualized using the LI-COR Odyssey software.

### Parent of origin determination of alleles at the *IGF2 *DMR2

To determine the allele origin of *IGF2 *DMR2 we used a single nucleotide polymorphism (SNPs) in the 3'-UTR at position *IGF2*-exon9-612A>T. Based on this SNP we analyzed parental DNA methylation patterns at the *IGF2 *DMR2.

## Authors' contributions

MHB conceived and designed the study. MOL sequenced the clones and performed the expression study. NSS isolated DNA and RNA and did the bisulfite analysis. MHB performed the 'hot-start' PCR, the methylation analysis and wrote the manuscript. All authors read and approved the final manuscript.

## Acknowledgements

We thank Leeson J. Alexander for careful reading the manuscript and critical comments. This research project has been co-financed by the European Commission, within the 6th Framework Programme, contract no. FOOD-CT-2006-016 250. The text represents the authors' views and does not necessarily represent a position of the Commission, who will not be liable for the use made of such information.

## Supplementary Material

Additional file 1 **Primer sequences, annealing temperatures and product sizes**. The table contains PCR primer sequence information including annealing temperatures and product sizes. Click here for file
