# Supplementary material for: Relationship of porcine IGF2 imprinting status to DNA methylation at the H19 DMD and the IGF2 DMRs 1 and 2
Source: BMC Genet. 2011 May 17;12:47. doi: 10.1186/1471-2156-12-47 (PMC3112248; doi:10.1186/1471-2156-12-47)
Supplement: Additional file 1 — Primer sequences, annealing temperatures and product sizes. The table contains PCR primer sequence information including annealing temperatures and product sizes. [file 1471-2156-12-47-S1.DOC]

Table S1. Primer sequences, annealing temperatures and product sizes.

| Name | Forward primer | Reverse primer | Annealing temperature | Product size |
| --- | --- | --- | --- | --- |
| IGF2_DMR1 | GAGGTTTAGGGGTTTAGATTTTTTT | ACCCAACATTTAACAAACCCAACTC | 57°C | 584 bp |
| IGF2_DMR2 | TATYGGAAGTGAGTTAAATTGT | AAAACCAAATTCTTTTATTTTACC | 56 and60°C | 470 bp |
| SWC9 | GGCTCAGGGATCCCACAG | AAGCACCTGTACCCACACG | 58°C | varies |
| H19_DMD_1 | TGGTTAGGGATAGGAGATTAGGTTTA | AATAACAACTACCACTCCCCTCATAC | 60°C | 492 bp |
| H19_DMD_2 | TTT TAG GTA TGA GGG GAG TGG TAG | CAA AAT AAC ACC TAA TAC TAA CAA C | 60°C | 687 bp |
| H19_DMD_3 | ATTATTAAGGTTGGGATTTGAGATT | aaccctataccaccaaaaaccctac | 60°C | 549 bp |
| H19_DMD_4 | GGGGTAAGGAGGGATTTTTATTTT | ATCCAAACCTAACCACACCTTAAAC | 60°C | 455 bp |
| hot-stop | GTAAAACGACGGCCAGATGTGGTAATG TGCCCTGTG | CAGATACGTGAGGCTGCACT | 60°C | 464 bp |
| M13 | IRD700GTAAAACGACGGCCAG |  | 60°C |  |
